# SGLT2i and GLP-1 RA therapy in type 1 diabetes and reno-vascular outcomes: a real-world study

**DOI:** 10.1007/s00125-023-05975-8

**Published:** 2023-07-28

**Authors:** Matthew Anson, Sizheng S. Zhao, Philip Austin, Gema H. Ibarburu, Rayaz A. Malik, Uazman Alam

**Affiliations:** 1 Diabetes & Endocrinology Research and Pain Research Institute, Institute of Life Course and Medical Sciences, University of Liverpool and Liverpool University Hospital NHS Foundation Trust, Liverpool, UK; 2 Centre for Epidemiology Versus Arthritis, Faculty of Biological Medicine and Health, The University of Manchester, Manchester Academic Health Science Centre, Manchester, UK; 3 TriNetX LLC, Cambridge, MA USA; 4 Weill Cornell Medicine-Qatar, Doha, Qatar; 5 Centre for Biomechanics and Rehabilitation Technologies, Staffordshire University, Stoke-on-Trent, UK

**Keywords:** Chronic kidney disease, Diabetic ketoacidosis, GLP-1 RA, Heart failure, SGLT2i, Type 1 diabetes

## Abstract

**Aims/hypothesis:**

Insulin is the primary treatment for type 1 diabetes. However, alternative glucose-lowering therapies are used adjunctively, but importantly are off-label in type 1 diabetes. Little work has previously been undertaken to evaluate safety with long-term efficacy and cardio-renal benefits of such therapies. We sought to investigate the real-world impact of sodium–glucose cotransporter 2 inhibitor (SGLT2i) and glucagon-like peptide-1 receptor agonist (GLP-1 RA) therapy in individuals with type 1 diabetes in relation to effect on blood glucose levels, adverse events and cardio-renal outcomes.

**Methods:**

We performed a retrospective cohort study of all patients aged 18 or over with type 1 diabetes on the TriNetX platform, a global collaborative network providing access to real-time, anonymised medical records. We included patients who had been treated with an SGLT2i or GLP-1 RA for at least 6 months and analysed the efficacy, safety and cardio-renal outcomes 5 years after initiation of therapy.

**Results:**

We identified 196,691 individuals with type 1 diabetes, 13% of whom were treated with adjunctive glucose-lowering therapy in addition to insulin. Included in the core analysis were 1822 patients treated with a GLP-1 RA and 992 individuals treated with an SGLT2i. Both agents provided clinically meaningful reductions in HbA_1c_ (−2.6 mmol/mol [−0.2%] with SGLT2i and −5.4 mmol/mol [−0.5%] with GLP-1 RA). The SGLT2i treated cohort showed preservation of eGFR over a 5-year period compared with the GLP-1 RA treated cohort (+3.5 ml/min per 1.73 m^2^ vs −7.2 ml/min per 1.73 m^2^, respectively), including patients with established chronic kidney disease (CKD). The SGLT2i treated cohort experienced higher rates of diabetic ketoacidosis (DKA) (RR 2.08 [95% CI 1.05, 4.12] *p*=0.0309) and urinary tract infection/pyelonephritis (RR 2.27 [95% CI 1.12, 4.55] *p*=0.019) compared with the GLP-1 RA treated cohort. However, the SGLT2i treated cohort were less likely to develop heart failure (RR 0.44 [95% CI 0.23, 0.83] *p*=0.0092), CKD (RR 0.49 [95% CI 0.28, 0.86] *p*=0.0118) and be hospitalised for any cause (RR 0.59 [95% CI 0.46, 0.76] *p≤*0.0001) when compared with the GLP-1 RA treated cohort.

**Conclusions/interpretation:**

Both SGLT2is and GLP-1 RAs have potential benefits as adjunctive agents in type 1 diabetes. SGLT2is provide cardio-renal benefits, despite an increase in the risk of DKA and urinary tract infection compared with GLP-1 RA therapy. Long-term evaluation of the efficacy and safety of these adjunctive therapies is required to guide their use in individuals with type 1 diabetes.

**Graphical Abstract:**

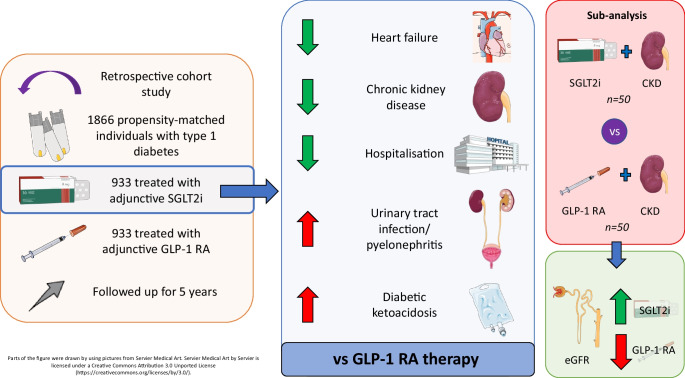

**Supplementary Information:**

The online version of this article (10.1007/s00125-023-05975-8) contains peer-reviewed but unedited supplementary material.


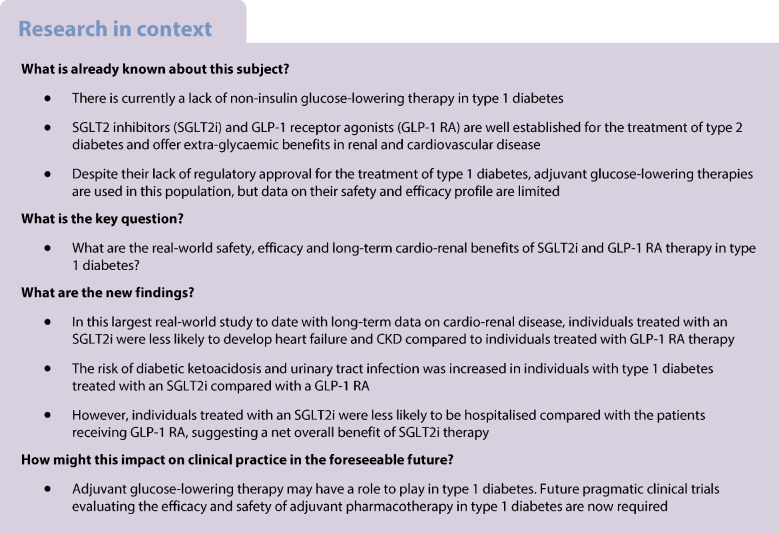


## Introduction

The incidence and prevalence of type 1 diabetes is increasing. It is estimated that 8.4 million individuals are living with the disease worldwide, and this is expected to increase [1]. Insulin has remained the cornerstone of treatment in type 1 diabetes since its discovery by Banting and Best in 1922 [2]. Despite advancements in ultra-fast and ultra-long-acting insulins, there has been a paucity of new non-insulin glucose-lowering therapies specifically licensed for type 1 diabetes.

Type 1 diabetes is unique amongst many of the chronic diseases where escalation of therapy does not routinely include the addition of alternative adjunctive pharmacotherapy. Intensification of glucose-lowering therapy in type 1 diabetes includes multiple daily insulin injections, continuous subcutaneous insulin infusion, alongside continuous glucose monitoring and increasingly closed-loop insulin delivery [3]. Pramlintide is an amylin analogue licensed for type 1 diabetes in the US but currently very few people are treated with it [4], and the risk of severe hypoglycaemia remains a barrier to its approval in the UK and Europe [5]. Early optimisation of glycaemic control is key to reducing the incidence of microvascular and macrovascular complications as demonstrated by the landmark DCCT [6]. However, the ‘ominous octet’ as described by DeFronzo, suggests that multiple drugs targeting different metabolic abnormalities in type 2 diabetes are required to reduce subsequent morbidity and mortality [7]. A similar rationale may also be applicable in type 1 diabetes.

Despite the advances in technology and insulin delivery over the past several decades, less than a third of adults with type 1 diabetes in England and Wales achieve a target HbA_1c_ of ≤58 mmol/mol [7.5%] [8], and only 20% of patients in the US T1D Exchange Registry achieved HbA_1c_ ≤53 mmol/mol [7.0%] [9]. Novel adjuvant therapies for type 1 diabetes have remained elusive.

The management of type 2 diabetes has included targeting multiple metabolic pathways to achieve optimal glycaemic control. Inhibitors of the sodium-glucose cotransporter 2 (SGLT2i) and glucagon-like peptide-1 receptor agonists (GLP-1 RA) have been studied and trialled in type 2 diabetes with an overwhelming body of evidence supporting their use in type 2 diabetes [10]. SGLT2is and GLP-1 RAs consistently demonstrate cardiovascular and nephroprotective effects [11]. SGLT2is, and GLP-1 RAs in particular promote weight loss, and therefore especially may be of benefit, given that overweight and obesity is increasingly prevalent (62%) in individuals with type 1 diabetes [12]. Furthermore, cardiovascular and renal disease are the predominant cause of premature mortality in type 1 diabetes [13]. Given that both SGLT2i and GLP-1 RA demonstrate robust and consistent cardio-renal benefits [14], both agents seem to be an attractive option to fulfil an unmet therapeutic need in type 1 diabetes. However, concerns regarding the safety of such agents, in particular the increased risk of diabetic ketoacidosis (DKA) with SGLT2is, have restricted their widespread adoption in type 1 diabetes [15]. Thus, all adjuvant glucose-lowering therapies in type 1 diabetes are used off-label and contrary to local and national guidance [16].

The administration of such agents could therefore be: (1) inappropriate and should not be prescribed off-label in type 1 diabetes; or (2) underutilised with a failure to capitalise on the significant glucose-lowering and cardio-renal benefits. This study has investigated the real-world impact of SGLT2i and GLP-1 RA therapy on individuals with type 1 diabetes in relation to efficacy, safety and cardio-renal outcomes.

## Methods

### Network characteristics

We performed a retrospective cohort study of all patients aged 18 or over with type 1 diabetes using the TriNetX platform. TriNetX is a global collaborative network which provides access to real-time electronic medical records (diagnoses, procedures, medications, laboratory values, genomic information) from approximately 125 million patients from 96 healthcare organisations (HCOs), primarily in North America and Western Europe. Data was collected from secondary care institutions. Specifically, for this retrospective cohort analysis, ~200,000 patients from 90 HCOs were identified. The data used in this study was collected on 17 January 2023. Further details can be found in electronic supplementary material (ESM) Methods.

### Building cohorts in TriNetX

Patients aged 18 or over with type 1 diabetes were identified based on the inclusion of the ICD-10-CM code E10 (http://apps.who.int/classifications/icd10/browse/2016/en) in their electronic medical record (EMR). To avoid the potential of individuals with type 2 diabetes, diabetes mellitus due to an underlying condition, drug or chemical induced diabetes and any other specified type of diabetes mellitus being included and skewing the analysis, the ICD codes E11, E08, E09 and E13, respectively, were used as exclusion criteria.

**Fig. 1:**
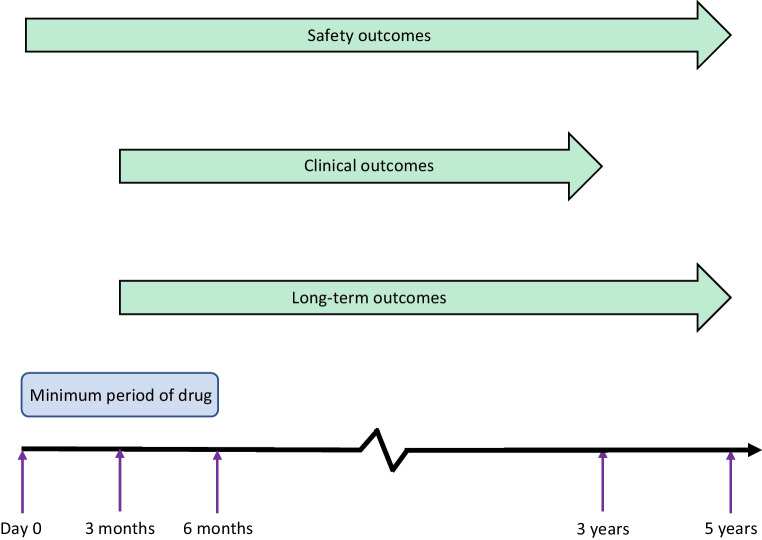
Summary of analysis time frame and inclusion criteria length

This initial group of participants were then divided into two cohorts, ‘SGLT2i’ and ‘GLP-1 RA’. We adopted an active comparator new user design where analysis was of new starters of each drug. For the SGLT2i cohort, we included patients who had received an SGLT2i for at least 6 months (Fig. 1), and who had a follow-up encounter with health services within a year post drug initiation. We excluded patients who had ever received a GLP-1 RA. For the GLP-1 RA cohort, we followed the same protocol as previously described, but excluded those who had ever been co-prescribed an SGLT2i at any time.

The analyses were conducted on the above cohorts: (1) SGLT2i and (2) GLP-1 RA with each analysis propensity score matched (PSM) for age, sex, BMI, presence of heart failure (ICD-10-CM-I50), hypertension (ICD-10-CM-I10), ischaemic heart disease (IHD) (ICD-10-CM-I20–25), chronic kidney disease (CKD) (ICD-10-CM-N18) and HbA_1c_ value so as to balance the analysis (1:1 matching) being undertaken. It was not possible to match HbA_1c_ to a mean value between cohorts; instead participants were matched into two HbA_1c_ categories: ≤53 mmol/mol (7%) and >53 mmol/mol (7%). We used ‘greedy nearest neighbour matching’ with a caliper of 0.1 pooled standard deviations. Any baseline characteristic with a strictly standardised mean difference (SSMD) <0.1 was considered to be well matched. Further detail of the algorithm can be found in ESM Methods.

The index event was defined as the initiation of an SGLT2i in the SGLT2i cohort and a GLP-1 RA in the GLP-1 RA cohort. The analysis on the respective cohorts was based on the following safety outcomes over 5 years from drug initiation: DKA, hypoglycaemia requiring admission to secondary care, urinary tract infection (UTI), pyelonephritis, candidiasis of the lower genital tract, acute pancreatitis and gastrointestinal upset. Individuals who developed a concerning adverse safety outcome were not censored and remained in any subsequent analysis. Clinical outcomes (change in HbA_1c_, weight, BMI, eGFR and cholesterol, compared with baseline) were recorded and analysed from 3 months to 3 years post index date. The first time a clinical outcome was recorded within the above time frame was counted in the analysis. Key end points included: hospitalisation, heart failure (ICD-10-CM-I50), myocardial infarction (ICD-10-CM-I21), stroke and transient ischaemic attack (ICD-10-CM-I63, G45), development of diabetic microvascular complications (ICD-10-CM-E10.2–10.4) and all-cause mortality analysed 3 months to 5 years post drug initiation. The 3 month lag was introduced for clinical and long-term outcomes to allow for an adequate amount of time to achieve some meaningful efficacy. In analysis of independent outcomes, individuals with a history of an outcome of interest prior to the drug initiation were excluded. Figure 1 summarises the time frames utilised in our study.

Statistical analysis was performed in situ within the TriNetX platform. Normally distributed baseline characteristics are presented as mean and SD. Risk ratio (RR; risk for cohort one/risk for cohort two) and 95% CIs are presented. The *t* test and *χ*^2^ statistical testing were conducted for differences in outcomes between cohorts. *p*<0.05 was statistically significant.

## Results

**Fig. 2:**
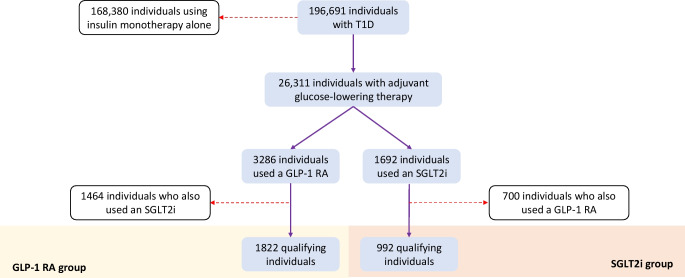
Breakdown of patient selection. T1D, type 1 diabetes

A total of 196,691 individuals with type 1 diabetes were identified. Of these, 87% were managed with insulin monotherapy alone, and 13% took additional glucose-lowering adjunctive therapy for glycaemic control. Metformin was the most common oral glucose-lowering therapy used, totalling 43% of all patients who were prescribed adjunctive therapies. A total of 3286 patients had taken a GLP-1 RA and 1692 had taken an SGLT2i at some point in time. A total of 992 patients were identified as using an SGLT2i without ever having been prescribed a GLP-1 RA. Similarly, 1822 patients had used a GLP-1 RA without ever having been prescribed an SGLT2i at any time. A breakdown of patient selection is summarised in Fig. 2. Within the GLP-1 RA cohort, 37% of individuals were treated with liraglutide, 25% with semaglutide, 24% with dulaglutide and 13% with exenatide. Within the SGLT2i cohort, 47% received empagliflozin, 27% received dapagliflozin and 25% received canagliflozin. Ten individuals in the SGLT2i cohort (<1%) and 27 individuals (<1%) in the GLP-1 RA cohort were also receiving pramlintide and were not specifically excluded from further analysis. Participants were followed up for a total of 5 years post drug initiation. Of the 1822 participants on a GLP-1 RA, 72% (1309 individuals) were still on therapy for at least 3 years. Of the 992 participants on an SGLT2i, 65% (647 individuals) were still on therapy for at least 3 years. Ninety-seven per cent of the individuals from the GLP-1 RA cohort were from the USA and 3% from the UK-EU. Of the individuals from the SGLT2i cohort, 87% were from the USA and 13% were from the UK-EU. Table 1 summarises the baseline characteristics of individuals and baseline demographics of the whole network are summarised in ESM Results.

**Table 1:** Baseline participant characteristics

Characteristic	SGLT2i (*n*=992)	GLP-1 RA (*n*=1822)
Age at index event (years)	53.3±15.4	47.1±15.8
Sex (male/female) (%)	53/47	37/63
Race (White/Black or African American/Asian/Native American/Unknown) (%)	68/7/4/2/19	74/10/1/1/14
Neuropathy (%)	3	3
Retinopathy (%)	4	4
Nephropathy (%)	4	3
Weight (kg)	91.2±21.9	99.3±24.4
BMI (kg/m^2^)	30.2±6.6	33.5±7.1
Systolic BP (mmHg)	130±20	129±18
Diastolic BP (mmHg)	75±11	77±10
eGFR (ml/min per 1.73 m^2^)	79.9±28.6	87.7±29.6
Cholesterol (mmol/l)	4.4±1.1	4.6±1.1
HbA_1c_ (mmol/mol)	65.0±20.9	62.8±19.8
HbA_1c_ (%)^a^	8.1±1.9	7.9±1.8
DKA history (%)	3	3
Heart failure (%)	10	3
Primary hypertension (%)	28	25
IHD (%)	18	11
CKD (%)	5	3

Data expressed as mean ± SD

^a^Original HbA_1c_ data presented in DCCT %, with conversion for mean HbA_1c_ in mmol/mol

Participants in the GLP-1 RA cohort were on average younger, more frequently female and had higher weight compared with those using an SGLT2i. At baseline, participants in the GLP-1 RA group had a preserved eGFR compared with those in the SGLT2i group and were less likely to have a diagnosis of hypertension, IHD and heart failure. The prevalence of diabetes-associated microvascular complications across both cohorts was low (3–4%).

After PSM, the number of participants in the SGLT2i group reduced from 992 to 933 and from 1822 to 933 in the GLP-1 RA group. Post PSM, participants in the GLP-1 RA group had a higher BMI and HbA_1c_ compared with the SGLT2i cohort. Groups were well matched for age, sex, baseline hypertension, heart failure, IHD and CKD. Table 2 summarises the propensity score matching characteristics.

**Table 2:** Baseline characteristics before and after propensity score matching

Characteristic	Before PSM			After PSM		
	SGLT2i (*n*=992)	GLP-1 RA (*n*=1822)	SSMD	SGLT2i (*n*=933)	GLP-1 RA (*n*=933)	SSMD
Age (years)	53.3±15.4	47.1±15.8	0.393	52.2±15.4	52.0±15.1	0.012
Sex (female) (%)	47	63	0.293	50	50	0.030
HbA_1c_ ≥53 mmol/mol (%)^a^	23	32	0.205	23	29	0.111
BMI (kg/m^2^)	30.2±6.6	33.5±7.1	0.482	30.2±6.6	33.1±6.85	0.388
Heart failure (%)	10	3	0.275	5	6	0.032
Primary hypertension (%)	28	25	0.069	27	25	0.064
IHD (%)	18	11	0.214	15	15	0.015
CKD (%)	5	3	0.095	4	4	0.051

Data expressed as mean ± SD

^a^Conversion to 7% in DCCT %

Participants using a GLP-1 RA had a greater reduction in HbA_1c_ −5.4 mmol/mol (−0.5%) vs −2.6 mmol/mol (−0.2%) and serum cholesterol (−0.4 mmol/l vs −0.1 mmol/l) than those in the SGLT2i cohort. Participants in the SGLT2i group demonstrated improvement in renal function with an increase in mean eGFR over the 5-year follow-up period (+3.5 ml/min per 1.73 m^2^), compared with an overall reduction in eGFR in the GLP-1 RA cohort (−7.2 ml/min per 1.73 m^2^). Interestingly, participants receiving GLP-1 RA therapy demonstrated an increase in weight compared with the SGLT2i group. Table 3 summarises key biochemical and anthropometric changes.

**Table 3:** Change in outcomes over 5 years

Characteristic	SGLT2i (*n*=933)			GLP−1 RA (*n*=933)		
	Baseline	Post initiation	Change	Baseline	Post initiation	Change
HbA_1c_ (mmol/mol)	65.0±20.9	62.8±17.6	−2.6	62.8±19.8	57.4±14.3	−5.4
HbA_1c_ (%)^a^	8.1±1.9	7.9±1.6	−0.2	7.9±1.8	7.4±1.3	−0.5
Weight (kg)	91.2±21.9	88.8±21.4	−2.4	99.3±24.4	100.8±27.0	+1.5
BMI (kg/m^2^)	30.2±6.6	30.0±5.9	−0.2	33.1±6.9	33.5±7.1	+0.4
eGFR (ml/min per 1.73m^2^)	79.9±28.6	83.4±28.3	+3.5	87.7±29.6	80.5±30.0	−7.2
Cholesterol (mmol/l)	4.4±1.1	4.3±1.1	−0.1	4.6±1.0	4.2±1.6	−0.4

^a^Original HbA_1c_ data presented in DCCT %, with conversion for mean HbA_1c_ in mmol/mol

Data expressed as mean ± SD

**Fig. 3:**
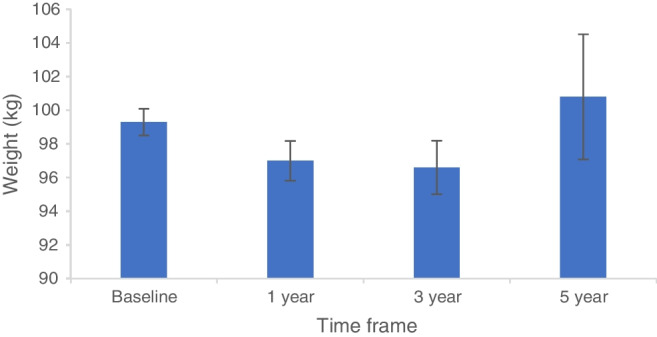
Graph demonstrating change in weight with GLP-1 RA use over time. Data are mean ± SEM

We performed time-based analysis on changes in body weight with GLP-1 RA as summarised in Fig. 3. Maximal weight loss was achieved at year 3 with a mean weight of 96.6±27.4 kg (−2.7% loss of baseline body weight) before rebounding and rising to 100.8±27.0 kg at year 5.

**Fig. 4:**
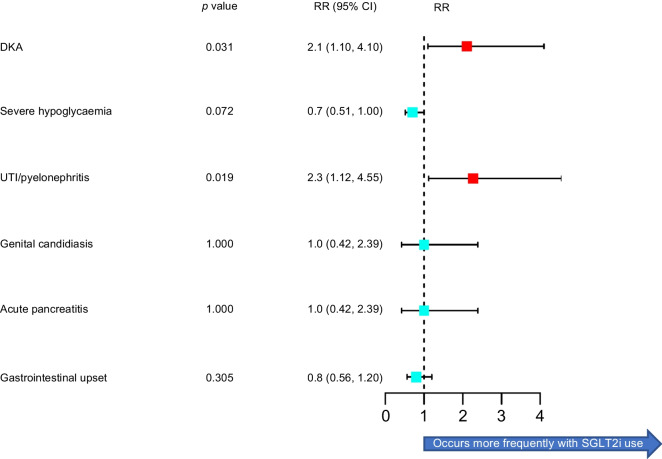
Three year risk of adverse events forest plot. Red boxes indicate statistically significant findings

SGLT2i use was associated with a significantly increased risk of DKA (RR 2.08 [95% CI 1.05, 4.12] *p*=0.0309). Over 5 years, 28 participants from the SGLT2i group developed DKA, compared with 11 in the GLP-1 RA group. All participants who had more than one episode of DKA were from the SGLT2i group. Those treated with an SGLT2i were more than twice as likely to develop a UTI or pyelonephritis than those treated with a GLP-1 RA (RR 2.27 [95% CI 1.12, 4.55] *p*=0.019). There was no difference in severe hypoglycaemia, genital candidiasis, acute pancreatitis, or gastrointestinal upset (nausea, vomiting and diarrhoea) between the two groups. Rates of severe hypoglycaemia (6%) and pancreatitis (1%) were no greater than in the general type 1 diabetes population (6% and 1%, respectively). Figure 4 summarises the 3-year adverse events. Absolute numbers of individuals with each outcome of interest are presented in ESM Table 1.

**Fig. 5:**
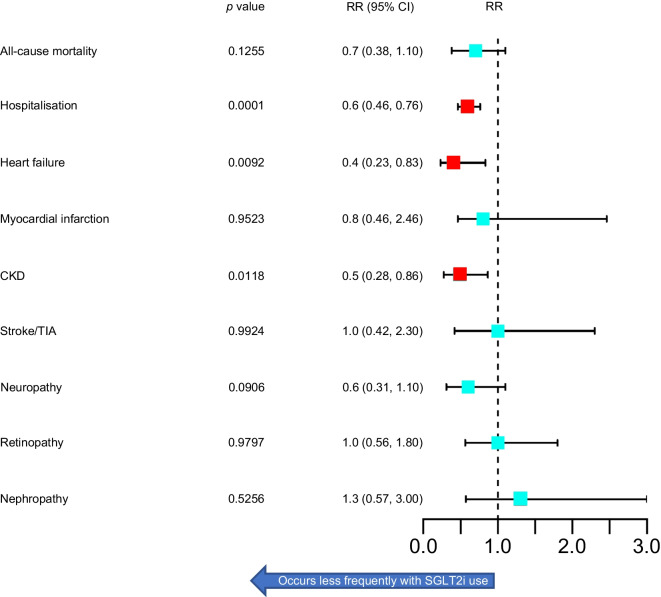
Five year outcomes forest plot: mortality, hospitalisation, and development of new macro- and microvascular outcomes during treatment. Red boxes indicate statistically significant findings. TIA, transient ischaemic attack

Seventy-four participants in each cohort were excluded for having a pre-existing diagnosis of heart failure. In the GLP-1 RA group, 33 participants were subsequently newly diagnosed with heart failure, compared with 15 in the SGLT2i group (RR 0.44 [95% CI 0.23, 0.83] *p*=0.0092). Participants in the SGLT2i group were less likely to require admission to hospital for any reason than those in the GLP-1 RA group (RR 0.59 [95% CI 0.46, 0.76] *p*≤0.0001). Patients treated with SGLT2i were less likely to be diagnosed with new CKD over a 5-year period post initiation compared with the GLP-1 RA cohort (RR 0.49 [95% CI 0.28, 0.86] *p*=0.0118). SGLT2i use was associated with less frequent hospitalisation and shorter duration of stay (*p*=0.0001). In the GLP-1 RA group, 133 participants were hospitalised (mean number of hospitalisations=9, median number of hospitalisations=3) over a 5-year period with a mean and median length of stay of 6 and 2 days, respectively. In the SGLT2i group, 101 participants were hospitalised (mean number of hospitalisations=2.7, median number of hospitalisations=2), with a mean and median length of stay of 2.4 and 2 days, respectively. There was no significant difference in all-cause mortality between cohorts. Figure 5 summarises the 5-year outcomes of interest.

**Fig. 6:**
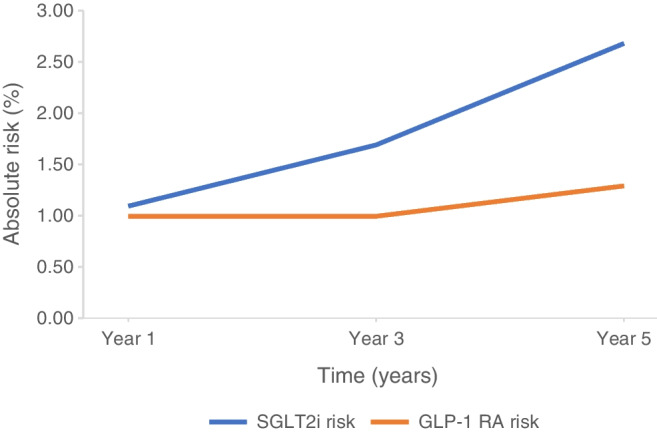
Graph demonstrating change in absolute risk over time of DKA between SGLT2i and GLP-1 RA cohorts

We performed additional time-based analysis for the risk of DKA between the two cohorts. In the GLP-1 RA cohort, the risk of DKA remained broadly constant, whilst the risk of DKA within the SGLT2i cohort increased with time and the difference in risk became statistically significant at year 5. Figure 6 demonstrates the risk of DKA over time.

We identified individuals within the SGLT2i and GLP-1 RA cohort who had a pre-existing diagnosis of CKD and conducted further subgroup analysis on this group over 5 years. We propensity matched for sex and presence or absence of IHD and heart failure. Baseline characteristics are shown in Table 4.

**Table 4:** Baseline characteristics of the subgroup with CKD

Characteristics	SGLT2i + CKD (*n*=50)	GLP-1 RA + CKD (*n*=50)	SSMD
Age at index event (years)	59.1±15.0	57.0±14.9	0.143
Sex (male/female) (%)	54/46	58/42	0.088
eGFR (ml/min per 1.73 m^2^)	45.5±16.7	50.1±23.1	0.231
IHD and/or heart failure (%)	34	37	0.040

Data expressed as mean ± SD

Individuals with CKD were on average older, more likely to be male and have a higher burden of cardiovascular disease than those in the main cohort. Within the SGLT2i + CKD group, eGFR improved from 45.5 ml/min per 1.73 m^2^ at baseline to 47.0 ml/min per 1.73 m^2^ at 5 years. Within the GLP-1 RA + CKD group, eGFR reduced from 50.1 ml/min per 1.73 m^2^ to 44.8 ml/min per 1.73 m^2^ at 5 years. No individuals developed DKA.

## Discussion

We have conducted the largest real-world retrospective cohort study to date investigating the safety and outcomes of individuals with type 1 diabetes using SGLT2i and GLP-1 RA therapy, and the first to report cardio-renal benefits. We demonstrate that both therapies offer clinically significant reductions in HbA_1c_ with no difference in 5-year all-cause mortality. SGLT2i use is associated with an increased risk of DKA and UTI, but confers several favourable outcomes, notably preservation of renal function, reduced risk of developing heart failure and CKD, and of hospitalisation, despite a higher prevalence of heart failure, hypertension, IHD and CKD at baseline. We demonstrate that within a subgroup of individuals with established CKD treated with an SGLT2i, eGFR remains preserved over 5 years compared to those treated with a GLP-1 RA. Participants in the GLP-1 RA cohort had a greater weight and a more preserved renal function at baseline. These data suggest that physicians may be preferentially selecting patients with type 1 diabetes thought to benefit most from either cardio-renal benefits of SGLT2i therapy or the weight reduction associated with GLP-1 RA, established in type 2 diabetes [17]. We also report that 13% of patients with type 1 diabetes in our registry use adjuvant glucose-lowering therapy in addition to insulin, significantly higher than the 5.4% reported from the T1D Exchange Registry in the US [18].

The increased risk of DKA in the SGLT2i group, and the associated hospitalisation, was not great enough to offset the increased rate of hospitalisation of any cause in the GLP-1 RA group. These findings suggest that SGLT2i therapy may overall have more benefit than GLP-1 RA therapy in type 1 diabetes.

The benefit of SGLT2i therapy beyond improved glycaemic control was first reported in the EMPA-REG study which reported a reduced incidence of major adverse cardiovascular events in patients with type 2 diabetes [19]. Subsequently, multiple large outcome trials of SGLT2is have demonstrated a reduction in all-cause mortality and hospitalisation for heart failure in patients with HFrEF [20] and HFpEF [21], irrespective of diabetes status. A meta-analysis of 13,275 patients further supports that SGLT2i therapy significantly reduces the risk of hospitalisation and all-cause mortality [22]. The benefits of SGLT2i therapy also extends to CKD where there is a significantly reduced risk of decline in eGFR and progression to end stage renal failure, regardless of presence or absence of type 2 diabetes [23]. Given that atherosclerotic cardiovascular disease and CKD are the leading causes of morbidity and mortality in type 1 diabetes [24], it is reassuring that we show that SGLT2i therapy also confers similar benefits in type 1 diabetes. A previous real-world study on SGLT2i therapy showed a promising reduction in HbA_1c_, weight and insulin requirements in type 1 diabetes but did not present long-term safety or reno-cardiovascular outcome data [25].

There is consistent evidence from RCTs and real-world studies that SGLT2i use in type 1 diabetes is associated with an increased risk of DKA [14]. Despite legitimate safety concerns and the lack of FDA (US Food and Drug Administration) approval for their use in type 1 diabetes, SGLT2i are used as adjunctive therapy in individuals with type 1 diabetes [18]. The UK’s National Institute for Health and Care Excellence and European Medicines Agency approved dapagliflozin as an adjunctive treatment of type 1 diabetes in 2019 and 2018, respectively [26, 27]. To mitigate the well-established risk of DKA, patients were required to have a total daily insulin requirement of ≥0.5 U/kg, undergo frequent home ketone monitoring and receive structured education [28]. However, in November 2021, approval was withdrawn after the marketing authorisation holder withdrew the indication for type 1 diabetes across Europe and the UK. This was notably not due to any new safety concerns and may potentially be driven by the manufacturer’s reluctance to introduce a ‘black triangle’ to ensure clinician and patient awareness of the increased monitoring requirements in type 1 diabetes [29].

Various guidelines have been proposed to mitigate the risk of adverse events, which includes avoiding initiation in those at extremes of age, recent history of DKA and frequent history of severe fungal infection [30]. SGLT2i therapy below the lowest approved dose may further reduce the risk of DKA [31]. There are currently an equal number of studies in SGLT2i and dual SGLT2i/SGLT1i therapy reporting both an increased [32–34] and decreased risk of DKA at maximal permitted doses [34–36] when compared with minimally licensed doses. The dose dependent relationship of SGLT2i and incidence of DKA remains unclear and further investigation is warranted. Concerns have been raised that the rate of DKA in real-world practice would surpass rates seen in closely supervised RCTs [37]. Whilst RCTs have reported DKA ranging from 0.8–4.3% [34], real-world rates of DKA have ranged from 0.0 [38]–3.5% [25] with one outlier of 12.5% [14]. We report a DKA rate at 5 years of 2.1%. Thus, the current literature does not support the concern of an increased risk of DKA in real-life practice, beyond levels seen in clinical trials. An initial analysis of the FDA adverse event reporting system (FAERS) data demonstrated no consistent trends in DKA with SGLT2i use over time [39], however an alternative cohort study of 50,220 individuals with type 2 diabetes showed the highest risk of DKA shortly after SGLT2i initiation, which subsequently reduced with time [40]. We demonstrate a positive association between length of time on therapy and DKA; further investigation is required to establish if this time-based association persists and remains significantly elevated above that of the baseline type 1 diabetes population. There is much debate surrounding the ‘tolerability’ of an increased risk of DKA, with comparisons being made with troglitazone and its subsequent removal from the market where the risk of fatal liver failure was greater than the current risk of DKA with SGLT2i use [31]. However, the long-term cardiovascular and renal benefits need to be carefully evaluated against the risk of DKA.

GLP-1 RAs promote weight loss [41], hence our findings of an increase in weight with GLP-1 RA therapy at 5 years was interesting. On further time-based analysis, we demonstrated a modest reduction in weight with GLP-1 RA use within the first 3 years (−2.7% of baseline body weight) before rebounding to above baseline at 5 years. Several factors could compound the disappointing degree of sustained weight loss seen in our cohort. Lower doses of GLP-1 RA are typically used for glycaemic control than in weight loss. Efficacy of GLP-1 RA at reducing weight is proportional to the higher baseline weight, i.e. greater degree of weight loss is seen when initiating from a higher starting weight [42]. A significant proportion of our cohort used dulaglutide (24%) and exenatide (13%), which are less efficacious for weight loss compared with liraglutide and semaglutide [43]. A proportion of participants also discontinued GLP-1 RA therapy after 3 years and it is well described that individuals can re-gain two-thirds of their initial weight loss upon cessation of GLP-1 RA [44]. We did, however, observe a −5.4 mmol/mol (−0.5%) reduction in HbA_1c_ in the GLP-1 RA cohort, similar to reports from randomised controlled trials [45, 46]. GLP-1 RA use in type 1 diabetes has been associated with an increased risk of symptomatic and asymptomatic hypoglycaemia [45]. Whilst day-to-day hypoglycaemia was not coded in our network, we employed hypoglycaemia requiring hospital admission as a surrogate outcome of severe hypoglycaemia and there was no difference in the incidence in our cohort compared to the general population with type 1 diabetes. We have not observed any difference in the rate of acute pancreatitis or gastrointestinal disturbance associated with GLP-1 RA compared to SGLT2i therapy and patients with type 1 diabetes. Only one previous study has investigated the effect of GLP-1 RA on lipid status in type 1 diabetes and found no difference compared with placebo [47]. We report a significant reduction in total cholesterol in individuals with type 1 diabetes treated with a GLP-1 RA. Given the relatively positive safety profile of GLP-1 RA with a reduction in weight and HbA_1c_, without an increased risk of DKA, they are an appealing adjunctive therapy for people with type 1 diabetes.

This study does, however, have limitations typical of real-world data and we recognise the early exploratory nature of our work would benefit from further dedicated longitudinal studies with more robust design methodology in order to confirm our findings. We relied on accurate coding of medical data by healthcare providers, and we are unaware of the precise method of diagnosis of type 1 diabetes. However, given our data are predominantly North American, we would expect the diagnosis to be based on clinical presentation and biochemistry/antibody testing. Additionally, the overwhelming majority of our data are from the US, which limits generalisability to the rest of the world. Due to a lack of coding in the dataset, we were unable to report more nuanced and specific metrics such as duration of diabetes, total daily dose of insulin, percentage time in range and reason for starting adjunctive therapy, all of which are clinically relevant. However, given the size of the network, we were able to identify and include 2814 individuals for analysis, the largest real-world observational study of GLP-1 RA and SGLT2i use in type 1 diabetes to date. We were unable to record the average length of time each drug was prescribed for, but more than half of each cohort were still on SGLT2i and GLP-1 RA therapy 3 years post initiation. We did not differentiate between different types and doses of drugs in the two groups. As this was a real-world study, we intended to capture the greatest amount of data possible and report findings as a total aggregate by drug class for simplicity. Minor adverse reactions such as UTIs and candidiasis may have been underreported as patients may present to health services such as primary care physicians or community pharmacies, which were not part of the network. We did not include individuals treated with insulin alone as an active comparator as it would introduce a significant amount of bias with the lack of a codable index event (i.e. initiation of a drug or a new diagnosis) in an ‘insulin-only’ arm, but this is an area of future study. Introducing a scale of disease severity such as the New York Heart Association (NYHA) classification for heart failure and degree of albuminuria in CKD may have further strengthened our propensity matching.

Studies to clarify the risk–benefit ratio of DKA and improved cardio-renal outcomes with SGLT2i use in type 1 diabetes are required to help inform future guidelines and standardise clinical practice. Stratifying by the presence or absence of endogenous C-peptide as a potential protective factor against DKA should also be investigated. Multiple studies have demonstrated beneficial reductions in weight and HbA_1c_ with short acting GLP-1 RA in individuals with overweight or obesity and type 1 diabetes [45–48]. However, there has been no study to date investigating longer acting GLP-1 RA in individuals without overweight or obesity [49]. The improvement in HbA_1c_ following GLP-1 RA may primarily be driven by weight loss [50], but the mechanism may be more nuanced in individuals with type 1 diabetes and normal body weight. The glucagonostatic characteristics of GLP-1 RA could prove to be more relevant to individuals with beta cell deficiency [51] which contrasts with type 2 diabetes where beta cell dysfunction and impaired incretin effect may dominate [52]. To what extent this relationship translates into alterations in HbA_1c_, time in range and total daily dose of insulin in patients with ideal body weight and type 1 diabetes is yet to be determined. The efficacy and safety of combination treatment with both GLP-1 RA and SGLT2i has not been studied to date in type 1 diabetes. Pivotal clinical trials in continuous ketone monitoring, akin to currently available continuous glucose monitoring, are taking place in 2023 [53]. If successful, future work investigating the combination of an SGLT2i with a dual continuous glucose and ketone sensor may allow individuals with type 1 diabetes to access SGLT2i therapy.

### Conclusion

We report that individuals with type 1 diabetes treated with SGLT2i therapy demonstrate preservation of renal function, reduced rates of hospitalisation and reduced risk of heart failure when compared with patients receiving GLP-1 RA. GLP-1 RA use is associated with a greater but moderate reduction in HbA_1c_ and lower risk of DKA when compared with SGLT2i therapy. Careful and targeted patient selection are required, alongside robust education and novel technologies to monitor glucose and ketone levels to mitigate the risks and enhance the beneficial effects of SGLT2i and GLP-1 RA therapy. Dedicated long-term trials investigating the benefits of such therapies on hospitalisation, major cardiovascular adverse events and all-cause mortality are required.

## Supplementary Information

The online version of this article (10.1007/s00125-023-05975-8) contains peer-reviewed but unedited supplementary material.

Supplementary file1 (PDF 104 KB)

## Data Availability

To gain access to the data in the TriNetX research network, a request can be made to TriNetX (https://live.trinetx.com), but costs may be incurred, a data sharing agreement would be necessary and no patient identifiable information can be obtained. No data from Liverpool University NHS Foundation Trust were utilised in this analysis.

## Abbreviations

CKD: Chronic kidney disease
DKA: Diabetic ketoacidosis
FDA: US Food and Drug Administration
GLP-1 RA: Glucagon-like peptide-1 receptor agonist
HCO: Healthcare organisation
IHD: Ischaemic heart disease
PSM: Propensity score matching
SGLT2i: Sodium-glucose cotransporter 2 inhibitor
SSMD: Strictly standardised mean difference
UTI: Urinary tract infection
