# Supplementary material for: SGLT2i and GLP-1 RA therapy in type 1 diabetes and reno-vascular outcomes: a real-world study
Source: Diabetologia. 2023 Jul 28;66(10):1869–81. doi: 10.1007/s00125-023-05975-8 (PMC10473989; doi:10.1007/s00125-023-05975-8)
Supplement: Supplementary file 1 — Supplementary file1 (PDF 104 KB) [file 125_2023_5975_MOESM1_ESM.pdf]

ESM Methods

Network characteristics

Data collection, processing, and transmission are performed in compliance with all Data Protection laws applicable to the contributing HCOs, including the EU Data Protection Law Regulation 2016/679, the General Data Protection Regulation on the protection of natural persons regarding the processing of personal data, and the Health Insurance Portability and Accountability Act, the US federal law which protects the privacy and security of healthcare data. The TriNetX Global Collaborative Network is a distributed network (with most HCOs located in the USA), and analytics are performed at the HCO with only aggregate results being surfaced and returned to the platform. Data usage and publication agreements are in place with all HCOs.

TriNetX, LLC is compliant with the Health Insurance Portability and Accountability Act (HIPAA), the US federal law which protects the privacy and security of healthcare data, and any additional data privacy regulations applicable to the contributing HCO. TriNetX is certified to the ISO 27001:2013 standard and maintains an Information Security Management System (ISMS) to ensure the protection of the healthcare data it has access to and to meet the requirements of the HIPAA Security Rule. Any data displayed on the TriNetX Platform in aggregate form, or any patient level data provided in a data set generated by the TriNetX Platform, only contains de-identified data as per the de-identification standard defined in Section 164.514(a) of the HIPAA Privacy Rule. The process by which the data is de-identified is attested to through a formal determination by a qualified expert as defined in Section 164.514(b)(1) of the HIPAA Privacy Rule. Because this study used only de-identified patient records and did not involve the collection, use, or transmittal of individually identifiable data, this study was exempted from Institutional Review Board approval.

Propensity score matching algorithm

1. For each patient in each cohort, the system computes both the outcome(s) of interest and values for each covariate.

2. These data form a matrix of covariate values for each

3. The system performs a logistic regression on the pooled matrices, to "predict" which cohort each patient originates from. The value of this model for a patient is that patient's predicted probability of being in the second cohort or "propensity score." The outcome variable was not a co-variate in the propensity model.

4. For each patient in the smaller cohort, the system chooses as match from the larger cohort (if any patients in the larger cohort are close enough). The pairs then form a subset of each cohort.

5. The system compares outcomes on these after matching subsets, rather than the original cohorts

ESM Results

124,649,524 individuals are included within the network. 54% are Female and 46% are Male. Mean age is 44±24 years. 40% of individuals are White, 5% are Black or African American, 3% are Asian with the remaining 48% unknown. Sex and Ethnicity are presumed to be self-reported by individuals. Socio-economic status is not coded for within the network.

ESM Table 1: Absolute number of individuals with each adverse safety outcome.

	SGLT2i (n=933)	GLP-1 RA (n=933)
DKA	25	12
Severe hypoglycaemia	50	69
UTI/ pyelonephritis	25	11
Genital candidiasis	10	10
Acute pancreatitis	10	10
Gastrointestinal upset	45	55
